# Macro, Micro, and Molecular. Changes of the Osteochondral Interface in Osteoarthritis Development

**DOI:** 10.3389/fcell.2021.659654

**Published:** 2021-05-10

**Authors:** Xiwei Fan, Xiaoxin Wu, Ross Crawford, Yin Xiao, Indira Prasadam

**Affiliations:** ^1^Faculty of Science and Engineering, School of Mechanical, Medical and Process Engineering, Institute of Health and Biomedical Innovation, Queensland University of Technology, Brisbane, QLD, Australia; ^2^Orthopaedic Department, The Prince Charles Hospital, Brisbane, QLD, Australia; ^3^Australia-China Centre for Tissue Engineering and Regenerative Medicine, Queensland University of Technology, Brisbane, QLD, Australia

**Keywords:** osteoarthritis, osteochondral junction, cartilage, hypertrophy, interaction, endochondral

## Abstract

Osteoarthritis (OA) is a long-term condition that causes joint pain and reduced movement. Notably, the same pathways governing cell growth, death, and differentiation during the growth and development of the body are also common drivers of OA. The osteochondral interface is a vital structure located between hyaline cartilage and subchondral bone. It plays a critical role in maintaining the physical and biological function, conveying joint mechanical stress, maintaining chondral microenvironment, as well as crosstalk and substance exchange through the osteochondral unit. In this review, we summarized the progress in research concerning the area of osteochondral junction, including its pathophysiological changes, molecular interactions, and signaling pathways that are related to the ultrastructure change. Multiple potential treatment options were also discussed in this review. A thorough understanding of these biological changes and molecular mechanisms in the pathologic process will advance our understanding of OA progression, and inform the development of effective therapeutics targeting OA.

## Introduction

Osteoarthritis (OA) is the most common joint disorder that affects more than 303 million people worldwide (James et al., 2018), characterized by a series of symptoms, including synovial inflammation, malacia, fibrillation, clefting, and degradation of articular cartilage, subchondral bone sclerosis, and outgrowth of osteophytes (Brandt, 1988; Hulth, 1993). The pathogenesis of OA involves crosstalk between bone, muscle, tendon, synovium, and fat pad. All the elements above take part in the integrated occurrence and development of the disease (Yuan et al., 2014). In particular, the osteochondral interface, which is the interface between hyaline cartilage and subchondral bone of the joint, was recently shown to increase molecular exchange, or ease of fluid transport, change of thickness, and neurovascular growth with increasing stages of OA (Findlay and Kuliwaba, 2016; Pouran et al., 2017; Qin et al., 2019), suggesting that the osteochondral interface is a region of active tissue remodeling during the disease process. Several studies have shown that the genes involved in normal bone development, such as endochondral ossification, chondrocyte hypertrophy, and joint formation are activated during the progression of OA (Lin et al., 2009; Onyekwelu et al., 2009; Saito et al., 2010; Singh et al., 2020). It is noteworthy that, in some cases, modification of these developmental genes can impact the severity of OA (Dou et al., 2020). A thorough understanding from a structural and developmental biology perspective may provide important insights into the mechanism behind disease etiology, which may contribute to the development of novel strategies in treating OA. In the present review, we will give an update on research concerning the osteochondral interface changes during OA, including its morphology, histology, molecular interaction, and signaling pathways.

## The Osteochondral Interface Is an Active Tissue Responsible for Maintaining the Joint Homeostasis

The growth plate, also known as the epiphyseal plate or physis, is the area of developing tissue near the ends of the long bones in children and adolescents. During the process of growth and development, the superficial layer of the proliferation zone, which is close to the articular surface, proliferates outwards. In this process, the sedimentation and absorption of calcium occur at a similar rate, so that the objective of growth and development is achieved. Mature bone does not absorb the sedimented calcium salt and forms the calcified cartilage zone (CCZ), a thin interlayer of hard tissue between the hyaline articular cartilage and the subchondral bone (Aghajanian and Mohan, 2018).

**FIGURE 1:**
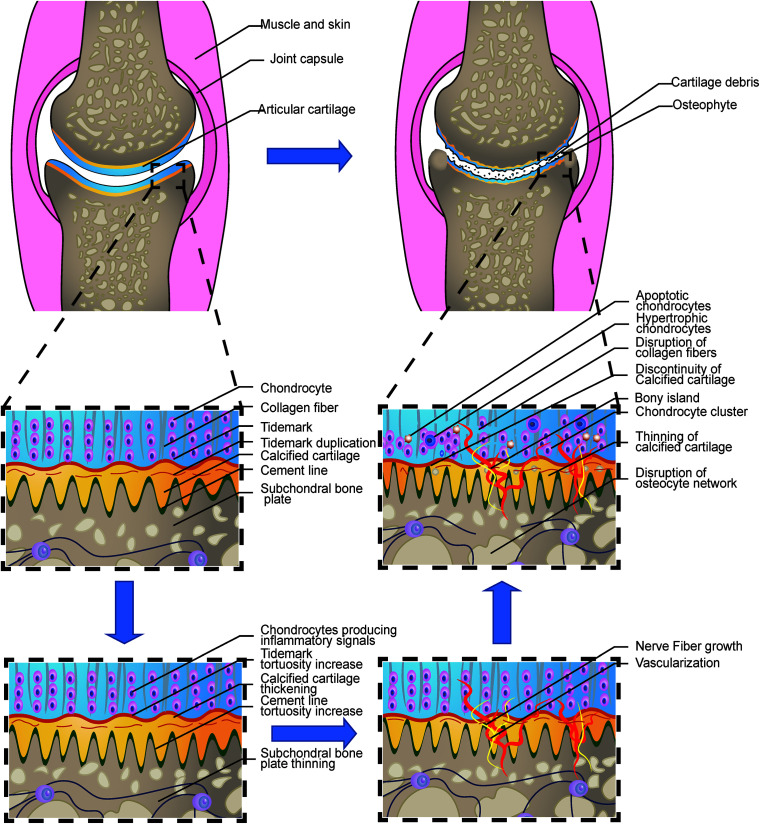
Loss of integrity in the osteochondral interface in osteoarthritis. The illustration shows the process from a normal healthy joint turning into the end-stage OA joint. The normal knee joint (left top picture) showed the integrated image of the joint. The articular cartilage covers the contact interface between the two adjacent joint surfaces. Chondrocytes are aligned properly in the three layers. The collagen fibers are intact and robust, perpendicular to the surface in the osteochondral junction in the deep layer. The thin layer of the CCZ is located beneath the hyaline cartilage. Tidemark duplication is presented in the tidemark. The cement line is wavier than the tidemark. Subchondral bone is located under the calcified cartilage layer. The osteocyte network is intact. In the early stage of OA, Chondrocytes produce multiple kinds of inflammatory signals. Cartilage swelling or edema is also common in the early stage. Chondrocyte proliferation begins in this stage. The subchondral bone remodeling also begins in this stage, resulting in the increased porosity of the subchondral bone plate. With the disease progression, the neurovascular invade the osteochondral interface. The tidemark and cement line expresses more tortuosity than before. And gradually, the collagen network in the cartilage is disrupted. Calcified cartilage thinning is common in the mid-late stage. Bone cyst and osteophyte formation are also common in the stage. And at the end stage, total breakage of the cartilage fibrin is commonly found. Hypertrophic and apoptotic cells are universally seen in the area. The bony island is seen in the calcified cartilage. Vascular elements and nerves grow into the uncalcified cartilage zone. Apoptotic osteocytes and disruption of the osteocyte network are the main change in the subchondral bone plate.

According to different concentrations in glycosaminoglycan, collagen, the orientation of the collagen fibers, and density of cells, the adult articular surface is separated into deep non-calcified cartilage, tidemark, CCZ, cement line, and the subchondral bone plate (Suri and Walsh, 2012; Figure 1). Among them, the non-calcified cartilage layer, also known as the hyaline cartilage layer, contains a superficial layer, a transitional layer, and a radiate layer (Benninghoff and Research, 1925; Lehner et al., 1989; Modl et al., 1991). The occurrence of tidemark is the sign of articular cartilage maturity. The CCZ connects the radiate layer with tidemark, which is the histologic division between the two layers. The calcified zone tightly connects the subchondral bone with the cement line. This close combination between the two layers dramatically increases the contact area and strength of the area. It also helps to disperse instantaneous stress to the subchondral bone (Wang et al., 2009; Suri and Walsh, 2012).

The osteochondral interface serves as the mechanical function as the wavy surface of the osteochondral interface tightly anchors the uncalcified cartilage to the subchondral bone. It also functions as the loading transition media from cartilage to the bone surface. The intermediate stiffness of CCZ facilitates the pressure conduction within the osteochondral interface, thus reducing the stress concentration due to the difference of the elastic module between the two materials. Under stressed conditions, the wavy contour and the collagen II in the osteochondral area can efficiently confront the shear force, thus safely conveying the pressure to CCZ. It is interesting to note that fractures often occur in the tidemark region in adults (Meachim and Bentley, 1978), however, in case of adolescents where there is still no clear separation in CCZ, the fracture tends to occur alongside the cement line and irregular surface of the subchondral bone (Matthewson and Dandy, 1978). This indicates CCZ plays an important role in preventing the elongation of cracks on the osteochondral interface.

The osteochondral zone also serves as the node for molecular transport, as well as crosstalk between uncalcified cartilage and subchondral bone. Earlier studies reported that the CCZ was incapable of transporting liquids and gas (Collins, 1949; Brower et al., 1962; Maroudas et al., 1968; Honner and Thompson, 1971; Ogata and Whiteside, 1979), suggesting that nutrients reached cartilage only through the articular surface via passive diffusion. However, this notion has recently been challenged by several lines of research. Pouran et al. (2017) tested equine and human samples using ultra-high micro-computerized tomography (micro-CT) and reported that decreased cartilage, CCZ, or subchondral bone thickness, as well as an increased porosity of subchondral plate or CCZ, are all positively correlated with diffusion transport. Multi-regression analysis of equine samples also showed a strong correlation between increased porosity, decreased thickness of subchondral bone or CCZ, and diffusion transport. However, these studies showed the correlation between porosity and diffusion in the equine sample but not in the human sample. Arkill and Winlove (2008) measured the diffusion speed quantitatively and reported that the CCZ of the horse was permeable to both fluorescein and rhodamine (m.w.∼400 Da), from both contact sides. The diffusion speed was 9 × 10^–9^ cm^2^ s^–1^, which is one-fifth of that in uncalcified cartilage. The effective diffusion coefficient of fluorescein in CCZ was 0.26 μm^2^/s, which was comparable to 0.9 μm^2^/s in horse calcified cartilage samples (Arkill and Winlove, 2008). Although the diffusion in the deep cartilage and subchondral bone is one hundred times slower than in the shallow layer of cartilage (Maroudas et al., 1968; Leddy and Guilak, 2003; Nimer et al., 2003), these small biomolecules are, however, infused very quickly throughout the joint. Arbabi et al. (2016) developed a finite elemental analysis, which confirmed the previous studies. An MRI imaging and human experiment also indicated that molecular signals could pass through the subchondral bone plate into the CCZ and hyaline cartilage (Berry et al., 1986; Imhof et al., 1999; Lyons et al., 2006).

## Zonal Properties of Calcified Cartilage and Deep Zone Cartilage Layer

### Normal Joint

In the deep uncalcified cartilage, several chondrocytes, with a spheroidal shape, accumulate to form a line that is perpendicular to the joint surface (Benninghoff and Research, 1925). Collagen fibers are denser and more tightly packed on the surface, while the collagen content is higher than that of the deep layer (Muir, 1995). CCZ zone is identified as a calcium mineralized cartilage layer located between the subchondral bone plate and the non-calcified hyaline cartilage (Ferguson et al., 2003; Frisbie et al., 2006). The difference in the degree of mineralization between CCZ and the subchondral bone plate is determined by the content of the extracellular matrix of cartilage (Frisbie et al., 2006). CCZ consists of sparsely distributed chondrocytes expressing a hypertrophic phenotype, which are arranged in columns, and are wrapped in a calcified hyaline cartilage matrix (Hunziker et al., 2002). The mineral content of CCZ is higher than that of the subchondral bone (Daley et al., 2019). The hypertrophic chondrocytes secrete matrix vesicles, alkaline phosphatase, and collagen X, which are responsible for mineralizing the extracellular matrix (Gannon et al., 1991; Hunziker, 1992). The extracellular matrix of the CCZ contains collagen types II and X, proteoglycan, and carbonated hydroxyapatite. The collagen fibers in CCZ are arranged perpendicular to the articular surface, passing through the tidemark, and connect with fibrils in the deep layer of articular cartilage. However, it is unclear whether these collagen fibrils pass through the cement line (Madry et al., 2010).

Quantitative analysis has shown that the mean, maximum, and minimum thickness of CCZ in the femoral condyle is 104.162 ± 0.87, 277.12 ± 8.6, and 9.83 ± 6.72 μm, respectively (Wang et al., 2009), which may be influenced by several factors. An increase in the thickness of CCZ has been reported to correlate with the duplication and thickening of tidemark, while a decrease in the thickness of CCZ is correlated with increased age and more loading bearing surface of the femoral head (Bullough, 2004). Interestingly, there is a sex-specific difference between males and females. Nielsen et al. (2019) analyzed the femoral heads of patients who had died accidentally and revealed that the thickness and volume of CCZ in the femoral head has a significantly negative correlation with age in females (*P* < 0.05) but not in males (*P* = 0.07 and 0.7, respectively). The cells are sparsely distributed in CCZ in the femoral condyle, most of which show a hypertrophic phenotype. The cell density of CCZ is ∼50 cells/mm2, which was lower than that of the uncalcified cartilage (*P* < 0.05) (Wang et al., 2009). Human-based research on the amino acid composition of the cartilage and X-ray diffraction (XRD) showed that, unlike uncalcified cartilage, type I collagen is the major component of the organic part. Type II collagen only accounts for ∼20% of CCZ in the medial femoral condyle. Hydroxyapatite remains the significant inorganic component, whereas its proportion (∼60%) in the total dry weight is less than that of subchondral bone (*P* < 0.05) (Zhang et al., 2012). The non-mineralized regions of the CCZ are composed of two distinct patterns, which include discrete larger non-mineralized plaques in denser deposits, and many smaller linear cavities within the whisker-like mineral deposits. Non-mineralized patches with linear distribution, ranging from 20 to 75 nm, are dispersed in randomly distributed dense deposits. In each of the denser deposits, a quasi-periodic mineralized fiber network and unmineralized spots and channels are shown, which are approximately 3–6 nm in diameter. The non-mineralized space took up ∼22% space of the total CCZ volume in the distal femur. Substances can be transported through these non-mineralized cavities, which may contain organic matter that is invisible under an electron microscope. The pore size for solute and fluid transport may be reduced due to the existence of organic matter (Oegema et al., 1997; Arkill and Winlove, 2008; Pan et al., 2009).

To date, studies on the gene expression profile in the CCZ layer are limited. The zonal difference of the uncalcified cartilage expression is observed in the cartilage (Grogan et al., 2013). Secreted Phosphoprotein 1 (SPP1) and Matrix Extracellular Phosphoglycoprotein (MEPE) are the two highly expressed genes in the deep zone cartilage compared with the superficial and intermediate cartilage zone. Furthermore, it has been reported that peroxisome proliferator-activated receptor gamma (PPARG) and epidermal growth factor receptor/silencing mediator for retinoid and thyroid hormone receptors-extended (EGFR/SMRTE) signaling pathway are enriched in the deep zone cartilage. PPARG signaling pathway is relevant to adipose and glucose metabolism, and EGFR/SMRTE signaling pathway is closely related to cell proliferation, differentiation, and apoptosis. In mature human cartilage, chondrocytes are the only cell type in the cartilage (Hunziker et al., 2014; Goldring and Goldring, 2016). In the deep non-calcified cartilage, the chondrocytes tend to form a line, following the collagen orientation. Although studies have reported that three subtypes, including pre-hypertrophic chondrocytes, hypertrophic chondrocytes, and proliferative chondrocytes exist in the growth plate (St-Jacques et al., 1999; Saito et al., 2010; Prein et al., 2016), new chondrocyte subtypes in mature cartilages were recently identified, including senescent cells and cartilage progenitor cells (Koelling et al., 2009; Jiang and Tuan, 2015; Jin et al., 2015; Worthley et al., 2015; Childs et al., 2017; Jeon et al., 2017). These cell subtypes contribute to tissue homeostasis in different ways. Senescent cells accumulate as people age and were found to be quiescent in the cell cycle (Jeon et al., 2017). While cartilage progenitor cells with self-renewal ability are capable of differentiating into chondrocytes, this subtype of the chondrocytes helps maintain cartilage repair and homeostasis in OA (Koelling et al., 2009; Jiang and Tuan, 2015).

### OA Joint

Although the increased thickness of CCZ has been associated with the progression of OA (Pritzker et al., 2006; Goldring and Goldring, 2016), several new studies have recently raised conflicting opinions. One study used anterior cruciate ligament dissection in the rat model and found that the tidemark roughness, rather than the CCZ area, is correlated with cartilage degeneration (Schultz et al., 2015). Another observational study revealed the CCZ change in human OA progression. In the early to medium stage of OA, the thickness of CCZ was generally increased (Bullough, 2004; Goldring, 2009), whereas the change of CCZ was different from that in the uncalcified cartilage or the subchondral bone. At the end stage of OA, the thickness of CCZ was decreased (Deng et al., 2016). The thickness change of the CCZ reflects the active remodeling process in OA. The rigorous mechanism involved in this process has not been determined precisely and may be related to the pro-angiogenic factors generated from chondrocytes in the radial layer of degenerative hyaline cartilage (Walsh et al., 2007). The microcracks and microfracture may also play essential but obscure roles in bone remodeling (Goldring, 2009). Intriguingly, the increased thickness of CCZ was also correlated with the area of vascular channel invasion, whereas the vascular channel number in this area was not relevant to the Osteoarthritis Research Society International (OARSI) grade or the thickness of CCZ. In the intermediate stage of OA, bone cover around the vessel was also found. Furthermore, the thickness of the subchondral bone was found to be correlated with the thickness of CCZ; the previous findings together indicated that these two tissues might function interactively in the OA (Deng et al., 2016). The discontinuity was also observed in the CCZ, thus resulting in the direct crosstalk between uncalcified cartilage and subchondral bone plate, as well as bringing about the changes in the molecular exchange (Lories and Luyten, 2011). The direct contact between bone and cartilage in the osteochondral interface deepens the interaction between chondrocytes and osteocytes and leads to holistic changes as the disease progress. It has been reported that the collagen of the deep zone cartilage is disrupted due to several important factors, including MMPs and other enzymes. The abundance of overall collagen content is decreased and the orientation of the collagen is disrupted (Goldring and Goldring, 2016). However, from the current literature, whether collagen change in the OA CCZ remains unknown. Chondrocyte density is also changed during OA progression. It has been reported that in the late stage, the chondrocyte density is decreased due to apoptosis, autophagy, and chronic low-grade inflammation (Loeser, 2013; Rahmati et al., 2017). Mineralization, fluid exchange, and porosity of CCZ are also changed during OA progression. Detailed changes are presented in the following sections.

In the mild or moderate stage of OA, the morphology and arrangement of the chondrocytes remain relatively unaffected (Pritzker et al., 2006). However, in the late OA stage, chondrocytes lose their line arrangement and form clusters. The chondrocytes density in the deep zone is also increased. This could be attributed to the activation and recruit of the chondrocyte progenitor cells (Lv et al., 2020). Gene expression varies greatly among different zones of the cartilage in OA progression. The deep zone chondrocytes expression undergoes some typical characteristic changes. Multiple gene expressions, including COL10A1, LECT1, MATN3, IBSP, and SPP1 are upregulated in the OA chondrocytes in the deep zone (Fukui et al., 2008b) indicating the endochondral ossification like signals are activated in this zone. Meanwhile, hypertrophic-related genes, including COL10A1 and WNT5B are also expressed characteristically in the deep zone. The expression of cartilage matrix genes, including type II collagen and aggrecan, are downregulated while the minor cartilaginous genes, including type III collagen and fibronectin, are upregulated (Fukui et al., 2008a). An overview of these changes is illustrated in Figure 1.

## Tidemark Region

### Normal Joint

In the original description of tidemark, Fawns and Landells considered the tidemark as an irregular basophilic line that can be stained with hematoxylin and eosin (Fawns and Landells, 1953; Simkin, 2012). Tidemark is also present in the unstressed areas but becomes wavy and thick in the stressed areas (Madry et al., 2010). Among the many theories of tidemark origin, one of the most popular is that it is the remains of the original growth plate (Simkin, 2012). This hypothesis is in line with the fact that the tidemark developed at the time and place where the growth plates accomplished their purpose. Tidemark is not seen in children but appears in all adults after puberty.

After maturity, the tidemark may not stay in its original position. New tidemarks often appear with changes in loading mechanics and the aging of OA joints. These new layers are like the original layer in every respect, but only one can mark the position where the original growth plate was located. Instead, reduplication must reflect an ongoing process (Broom and Poole, 1982; Havelka et al., 1984; Simkin, 2012). Tidemark thicknesses were ∼9 mm in the load-bearing areas and ∼4 mm in non-stressed regions. Age is not correlated with the thickness in stressed and non-stressed areas (*P* > 0.05) (Chen et al., 2011). However, a study of the femoral head shows that after the age of 65, the numbers and thickness of tidemark will be increased regardless of the stressed or non-stressed areas of the joint (Bullough, 2004). Sex is also an influencing factor. The surface area of the tidemark in males is remarkably more extensive than that of females (*P* < 0.05) (Nielsen et al., 2019). The duplication of tidemark is correlated with the attenuation of the entire articular cartilage. The tidemark is also an essential structure that binds cartilage with CCZ. A scanning electron microscope (SEM) study of human mandibular condylar cartilage reported that the uncalcified cartilage was connected with CCZ by collagen fibrils, which were shaped in a specific gradient (Chen et al., 2011). A histochemistry study indicated that there are three laminae in the tidemark. Two-dimensional material property maps also showed that a wavy band with a relatively low elastic modulus exists between CCZ and hyaline cartilage. Moreover, the superficial and the deeper layer are parallel with each other (Gupta et al., 2005). Some studies held the view that tidemark is made of two layers. Histological staining differences indicate that superficial and deeper layers of tidemark differ in their chemical properties. However, the central lamina tends to have a mixture of both layers according to its tinctorial nature. This phenomenon gives the impression that the proximal and distal layers may partially interpenetrate to form a central lamina, but there is no dyeable component that is not exhibited by at least one of the other two laminae, so it is hard to distinguish between the possible laminaes. Although cells were often seen to be closely adjacent to the tidemark, no cells were found embedded in the tidemark (Lyons et al., 2005). This finding corresponds to those of Bullough and Jagannath (1983) that chondrocytes were observed partly wrapped in the tidemark mineralized surface, but were not entirely sealed within it. Another study focusing on the osteochondral interface showed that the most significant differences of indentation modulus, hardness, and mean calcium content lies between hyaline cartilage and CCZ, which is much more significant than that of the differences between CCZ and the subchondral bone plate, implying a vital but obscure role of tidemark (Gupta et al., 2005). Surprisingly, the toxic element lead was found to has a high affinity to the tidemark (Zoeger et al., 2006), but the mechanism behind its accumulation in the tidemark remains unknown.

### OA Joint

The classical theory tends to correlate the duplication of tidemark with the progression of OA (Hsia et al., 2013). However, a recent study revealed that tidemark duplication does not only appear in OA, but it was also found in a healthy joint. Moreover, the number of tidemark duplication does not correlate with the OARSI grade or the thickness of CCZ (*P* > 0.05). Nonetheless, the level of roughness did have a relationship with OARSI and the thickness of CCZ (*P* < 0.05) (Deng et al., 2016). This is in accordance with the high-frequency ultrasound imaging finding (Huang et al., 2018). These findings indicate a new approach to view the changes in OA (Figure 1). The neurovascular invasion is an important pathological feature of OA. Several factors were involved in the vascular invasion through the tidemark, including the migration and infiltration of the macrophages, upregulation of the angiogenesis factors including VEGF, and the localization of the anti-angiogenesis factors in the superficial region, rather than the deeper osteochondral interface (Mapp and Walsh, 2012).

## Structural and Functional Properties of the Cement Line

Studies on the composition of the cement line are limited. The 3D reconstruction using a combination of serial sections of samples and Rhino 4.0 image analysis software showed that the cement line at their interface conjunction structure resembled a comb-like structure in longitudinal sections, and it formed an irregular arc path in cross-section (Wang et al., 2009). In one study, SEM showed that the cement line is topographically indistinct from the surrounding subchondral bone. A thorough demarcation was observed between collagen I and II at the cement line using lower magnification SEM and light microscopy (Gupta et al., 2005), which corresponds to Zizak’s study (Zizak et al., 2003). This disruption of fibers at the cement line suggested that cartilage and bone are attached only by mechanical interlocking due to the interdigitation of the CCZ and bone (Oegema et al., 1997). However, through fluorescence microscopy, it was recently reported that some collagen fibers were found penetrating both the subchondral bone and CCZ in horse osteochondral samples (Mansfield and Winlove, 2012). By using the high-resolution second-harmonic generation (SHG) imaging method, it was also revealed that collagen fibers are present across the cement line. Notwithstanding, the distribution of collagen was not even, and a non-fiber area was seen on a 50 μm scale. This study also indicated that the fiber content of collagen I and II differs between bone and cartilage, with the presence of the brighter image in the bone area on SHG. Subchondral bone was also found to contain thicker collagen fibers (Mansfield and Winlove, 2012).

## Structural and Functional Properties of the Subchondral Bone Plate

### Normal Joint

The subchondral bone plate is a part of the periarticular bone, which also contains the trabecular bone and joint edge bone (Goldring and Goldring, 2010; Hu et al., 2020). The main composition of the subchondral bone plate is cortical bone, which is poor neuro-vasculature and non-porous (Burr, 2004). The mean thickness of the subchondral bone plate is 216 ± 68–229 ± 52 μm in females and males, respectively. Hydroxyapatite accounts for most of the inorganic substances with ∼80% in dry weight (Zhang et al., 2012). The thickness and volume of the subchondral bone plate do not vary according to age or sex differences (Nielsen et al., 2016).

### OA Joint

Subchondral bone undergoes noticeable morphological changes throughout the OA process. Bone loss was found in the early stage of the disease process (Madry et al., 2016), followed by increased trabecular thickness, and finally cancellous bone collapse in the late stages (Barr et al., 2015). A thin subchondral plate has traditionally been associated with end-stage disease. However, there is evidence that subchondral bone thinning may be related to aging and OA (Yamada et al., 2002). Several studies reported that the subchondral bone plate changes more rapidly than trabecular bone in OA (Zamli et al., 2016). In the early OA, both the thickness and porosity of the subchondral bone plate are increased (Grynpas et al., 1991; Li et al., 1999; Palmer et al., 2013) accompanied by the reduced mineralization of the subchondral bone, and altered trabecular integrity (Kraus et al., 2013). Interestingly, this pattern co-exists with areas of articular cartilage damage (Iijima et al., 2016).

Unlike cartilage, subchondral bone quickly responds to changing mechanical force and rebalances the physiologic status through bone remodeling (Goldring and Goldring, 2016). Bone remodeling functionally allows bone tissue to adapt to changing mechanical forces and metabolic needs. However, it is also the basis of OA and many other bone diseases (Fowler et al., 2017; Stewart and Kawcak, 2018). Under normal physiological conditions, bone remodeling of subchondral bone maintains a dynamic balance through the osteogenic activity of osteoblasts and osteoclast degradation activity. During OA development, as the dynamic balance is destroyed, the metabolic activity of osteoblasts changes, and the subchondral bone undergoes structural changes (Lajeunesse and Reboul, 2003; Donell, 2019). The progression of OA cartilage degradation is closely related to bone remodeling and sclerosis of subchondral bone. The bidirectional conversion and the changing speed of bone remodeling were found in the subchondral bone (Maruotti et al., 2017). Subchondral bone remodeling in OA includes increased early bone turnover, microfracture, and later neovascularization and osteosclerosis. In the early stages of OA, subchondral bone resorption occurs, bone remodeling rate is increased (Findlay and Atkins, 2014), the bone turnover rate in the bone remodeling site is increased, and the subchondral bone plate thickness is decreased (Burr and Gallant, 2012). In the late stage of OA, bone resorption is reduced, and bone formation is increased (Prasadam, 2009). The occurrence of microcracks or microfractures has not been fully understood yet. In immature joints, fractures are frequent in the subchondral bone, while in mature joints, tidemark is the priority for microcracks (Broom et al., 1996). Therefore, the microcracks were found not only in the CCZ but also in the subchondral bone plate (Kraus et al., 2013; Iijima et al., 2016). These microcracks could be removed, repaired, and refreshed owing to the osteoclasts periodically (Iijima et al., 2016; Fowler et al., 2017). Earlier studies suggest that microcracks are a sign of OA. It was hypothesized that microcracks initiated OA by targeted remodeling, featured by the repair of microcracks in the CCZ (Goldring, 2009). More recently, microcracks have been reported to have a negative correlation with the progression of OA, indicating that microcracks are a necessity in the homeostasis of the cartilage-bone unit (Zarka et al., 2019). Based on previous findings, some researchers attribute the microcracks as the canals for osteoclast-chondrocyte crosstalk, the regulation of chondrocytes by osteoclast in the subchondral bone promotes the loss of cartilage integrity and OA progression (Hu et al., 2020).

Multiple signaling pathways are related to subchondral bone remodeling, mainly including TGF- β/Smad signaling pathway, and MAPK signaling pathway (Zhou et al., 2020). By simulating osteoclastogenesis, the excessive TGF-β leads to abnormal bone remodeling processes, including enhanced bone resorption, increased porosity of subchondral bone plate, and vasculature growth. In the progression of OA, bone remodeling of subchondral bone results from abnormal osteogenesis, which is caused by the excessive release of TGF-β which is related to abnormal mechanical stimulation (Zhen et al., 2013). The use of TGF-β-neutralizing antibody attenuates OA progression and preserves the subchondral bone microarchitecture (Xie et al., 2016). Excessive TGF-β can also result in the heterotopic ossification of subchondral bone (Tanamas et al., 2010). This is attributed to the disruption of the coupled bone remodeling by upregulated TGF-β. As a result, bone mesenchymal stem cells are recruited and differentiated in the bone marrow rather than the bone surface. Inhibition of the TGF-β can also inhibit the heterotopic ossification (Wang et al., 2018). In contrast, the MAPK signaling pathway meditates the subchondral bone formation by regulating the osteoblasts anabolism. Under appropriate mechanical loading, the MAPK signaling pathway and the classic Wnt/β-catenin signaling pathway promote the expression (Robinson et al., 2006). The overexpression of MAPK is closely correlated with the expression of MMPs in the osteoblasts, which can cleave the collagen triple helix domain, thus resulting in damage to the subchondral bone (Sondergaard et al., 2010).

## Endochondral Ossification Like Events in the Osteochondral Junction of OA Joints

The growth plate, also defined as the epiphyseal plate or physis, is the area of developing tissue near the ends of the long bones in children and adolescents. The endochondral ossification occurs in the bone-forming growth plates of children and adolescents. Epiphyseal cartilage tissue is ossified around the primary and secondary ossification center. During growth and development, the surface layer of the proliferation zone near the joint surface proliferates outward. Meanwhile, chondrocytes undergo terminal differentiation to hypertrophic chondrocytes and have the capacity to mineralize their ECM. The fate of the hypertrophic chondrocytes remains controversial. Previous studies indicated that the hypertrophic chondrocytes undergo apoptosis and osteoblasts proliferate to form bone (Farnum and Wilsman, 1987; Aizawa et al., 1997; Kronenberg, 2003; Mackie et al., 2011). However, recent studies using cell lineage tracing held a different view. When hypertrophic cartilage becomes calcified, the invasion process of blood vessels causes chondrocytes to transdifferentiate into osteoblasts in three ways, including direct trans-differentiation, dedifferentiation to redifferentiation, and chondrocyte to the osteogenic precursors (Aghajanian and Mohan, 2018), thereby remodeling the calcified cartilage template into the bone. In this process, the sedimentation and absorption of calcium occur at a similar rate, so that the objective of growth and development is achieved.

Multiple features demonstrate the similarity between the growth plate and the change of OA progression. In the growth plate, the endochondral ossification events, including sequential proliferation, chondrocyte hypertrophy, angiogenesis, apoptosis, and ossification, ensures the growth and maturity of the normal joint. The chondrocytes are aligned sequentially during this differentiation process. Several signaling markers are involved in this co-ordinated process, including Wnt and Notch signaling pathways, fibroblast growth factors (FGF), insulin-like growth factor (IGF-1), Indian Hedgehog (Ihh), and parathyroid hormone-related protein (PTHrP) (Lamuedra et al., 2020). In OA progression, the loss of integrity and remodeling of the cartilage leads to recurrence of the developmental genes, thus resulting in the endochondral ossification-like events in the uncalcified cartilage as well as in the CCZ layer. Multiple forms of endochondral ossification were reported during OA development, including the chondrocalcinosis in the hyaline cartilage, CCZ, and osteophyte formation. The occurrence of chondrocalcinosis in the OA hyaline cartilage has been widely reported (Fuerst et al., 2009; Xiao and Lin, 2016) and has a strong relationship with OA of the knee (Wang et al., 2019). Some researchers believe that the most direct expression of endochondral ossification is the thickening of CCZ, which leads to the thinning of hyaline cartilage (Xiao and Lin, 2016). Microcracks both in CCZ and subchondral bone are common occurrences (Radin and Rose, 1986; Burr and Radin, 2003) and these cracks were shown to be spontaneously repaired by forming a high-density mineralized infill (HDMI) phase. These protrusions were first found in the uncalcified cartilage tissue samples in racehorses (Boyde et al., 2011), and partial decalcified sections justified their existence (Turley et al., 2014). HDMI is always generated from the microcracks from CCZ and subchondral bone. Moreover, it can grow up to two-thirds of the thickness of the articular cartilage. These protrusions were made up of high mineralized compositions, as described by Boyde (2003). Similar findings were also found in the Icelandic horse, which is a model animal for OA (Ley et al., 2014). Recently, one paper reported the occurrence of the bony island in human hip joints (Boyde et al., 2014). The osteophyte is another important feature of OA. Endochondral ossification induced by hypertrophic chondrocytes is an important process for the formation of OA osteophytes. The hypertrophic chondrocytes inside the developing osteophytes will first undergo cartilage formation and accumulation of proteoglycans and other cartilage matrix-related factors. The fibroblast-like cells in the surface layer proliferate and differentiate to form chondrocytes. Hypertrophic chondrocytes present at the center further differentiate and form a bone marrow cavity through endochondral osteogenesis. The fully developed osteophytes fuse with the original subchondral bone. Eventually, the outer fibrous layer still exists, which is covered by cartilage and expands the original joint range. However, the mechanical properties of this kind of osteophytes are far lower than the original joints because they were not stimulated by the correct mechanical loading (Wong et al., 2016).

On the other hand, several research reports suggest OA cartilage displays the altered expression of mineralized related markers such as Osterix (Fuerst et al., 2009; Pulsatelli et al., 2013), RUNX2, Collagen X, and HIF-2α. In healthy joints, chondrocytes continuously release ATP (Graff et al., 2000), maintaining a high level of nucleotide pyrophosphatase-1 (NPP1) activity, and steadily secrete a significant amount of extracellular matrix and pyrophosphate (Johnson et al., 1999; Johnson and Terkeltaub, 2005). NPP1, which is correlated with downregulation of the bone formation, was found to decrease with the progression of OA (Bertrand et al., 2012). One study also indicated that NPP1 polymorphism was associated with hand OA (Suk et al., 2005), indicating the gene could potentially function in OA. The OA-like calcification pattern was found in the cartilage of mice as well (Sakamoto et al., 1994; Bertrand et al., 2012). Accordingly, NPP1 and pyrophosphate may play an essential but undefined role in downregulating calcification in OA as well as other bone diseases. All these features indicate the mineralization of articular cartilage is a common event in OA disease progression. There is a significant correlation between clinical symptoms and the amount of mineralized cartilage in OA.

## Hypertrophic Chondrocytes in OA CCZ Resemble the Hypertrophic Chondrocytes in Growth Plate

**FIGURE 2:**
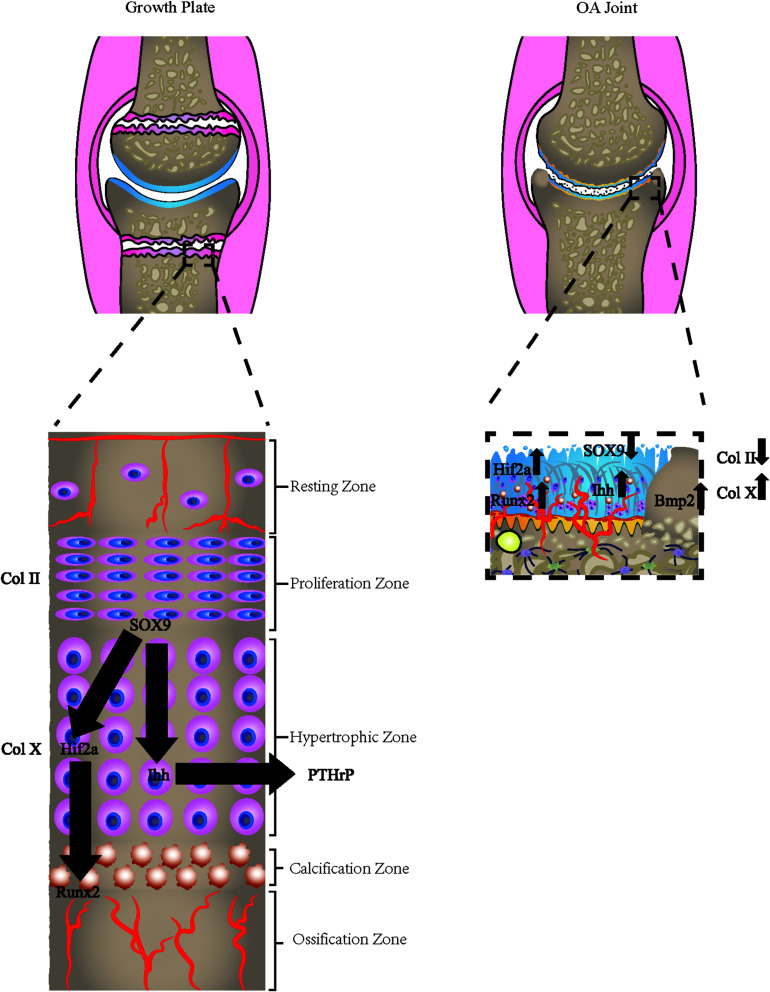
Osteoarthritic cells express growth plate signals. The osteoarthritic chondrocytes express hypertrophic chondrocyte phenotype, which is commonly found in the growth plate of adolescents. The hypertrophic markers, including Col X, HIF-2α, Runx2, Osterix, and relevant hypertrophic genes are all altered in the process.

Hypertrophic chondrocytes are often observed in the layers of long bone growth plates. Besides secreting specific proteins, such as collagen X, these cells also experience programmed apoptosis, making bone mineral and matrix deposition possible (Zenmyo et al., 1996; Billinghurst et al., 1997; Shlopov et al., 1997, 2000; Figure 2). The hypertrophic chondrocytes are not usually found in healthy knee cartilage. However, the expression of the hypertrophic markers and changes in morphology into hypertrophic phenotype has been observed in OA chondrocytes in both deep layers of cartilage and CCZ. The phenotypic changes may be the result of articular chondrocytes adopting a differentiation pathway similar to growth plate chondrocytes and expressing hypertrophic changes (van der Kraan and van den Berg, 2012). Another hypothesis is that after articular chondrocytes are dedifferentiated, they exhibit similar behaviors as terminally differentiated chondrocytes (hypertrophic), which are commonly observed on the growth plates of growing individuals (von der Mark et al., 1992; Dreier, 2010). Gene knockout and transgenic animal model studies have increasingly supported the role of chondrocyte terminal differentiation in the progression of OA. Genetic modifications that stimulate changes in chondrocyte hypertrophy are more likely to be associated with a higher incidence of OA or accelerated development of OA (van der Kraan and van den Berg, 2012).

Type X collagen is the golden standard for chondrocyte hypertrophy. It is usually expressed in hypertrophic areas of the epiphyseal plate. However, its expression levels are significantly upregulated in the protein and mRNA in OA human cartilage. Other markers of hypertrophy, collagenase-3 or Matrix metalloproteinase-13 (MMP-13), are also closely related to hypertrophic chondrocytes in OA (Moldovan et al., 1997). Chondrocyte hypertrophy is not strictly controlled by a single transcription factor but appears to be regulated by a regulatory system. Several transcription factors are involved in the transition to chondrocyte hypertrophy, including Runx2 (Higashikawa et al., 2009), NFAT1 (Caldwell and Wang, 2015), HIF-2α (Markway et al., 2013), β-catenin (Yang et al., 2020), Smad2/3 (Alvarez and Serra, 2004), Smad1/5/8 (Nishida et al., 2013), Interleukin-8 (Pesesse et al., 2014), and many other factors. Interestingly, all these factors are reported to be reactivated in OA development, suggesting that the changes in the chondrocyte phenotype mimic endochondral ossification signals. OPG and RANKL are also important factors for the hypertrophic chondrocytes. OPG knockout mice develop severe degenerative polyarticular disease, forming a thinner cartilage layer, accompanied by the gradual loss of cartilage matrix (Bolon et al., 2015). The study also found that cartilage in the mice model has low proliferation capacity and high cell apoptosis rate, with the low content of Type I and II collagen but high content of type X collagen (Upton et al., 2012). The high RANKL/OPG ratio is related to the up-regulation of catabolism (Kovács et al., 2019). According to reports, the RANKL/OPG ratio is first increased in early OA and then downregulated in late OA (Upton et al., 2012). This is in accordance with the bone absorption of subchondral bone in the early stage and bone formation in the late stage.

Multiple important signaling pathways related to the growth plate have been demonstrated to play important roles in OA. However, the osteochondral interface, which is the remains of the growth plate, and its relationship with the developmental genes have not been thoroughly studied. During the endochondral ossification process, the generation and dissolution of cartilage tissue are tightly regulated by multiple growth factors and hormones, among which EGFR signaling has been extensively studied in the past two decades. It is reported that the EGFR signaling pathway stimulates the extracellular matrix degradation to transform from cartilage to bone (Qin and Beier, 2019). The deficit EGFR mice were reported to suffer from delayed primary ossification center formation together with an expanded growth plate during early skeletal development, displaying elongated epiphyseal growth plates with a major expansion of the hypertrophic cartilage zone. EGFR signaling is important for RANKL expression in the growth plate and thus is responsible for osteoclastogenesis at the osteochondral interface (Zhang et al., 2011). Most studies on EGFR signaling in OA have concluded that, like some other growth factor signaling pathways, EGFR plays a dual role in articular cartilage. On the one hand, it has the effect of stimulating anabolism by stimulating proliferation and survival. Since proliferation is minimal in adult cartilage, this makes it an important factor for cartilage maintenance. Moreover, the EGFR signaling pathway can improve the lubrication function of the joint surface. This lubrication function can prevent joint damage in the early stage of OA (Jia et al., 2016). On the other hand, the EGFR signaling pathway also promotes catabolism and synthesis of tissue cartilage matrix (such as type II collagen and proteoglycans) by inhibiting the Sox9 expression. Besides, EGFR also promotes the activity of metalloproteinase matrix-degrading enzymes (Sun et al., 2018). In summary, the final effect of EGFR on the body is related to the balance of the above two effects, which is related to age, OA stage, and microenvironment. Further relationship between EGRF and osteochondral interface should be investigated carefully.

In both embryonic and adult tissues, GDF5 regulates chondrogenic cell growth and differentiation, and transgenic mouse studies suggest that GDF5 promotes differentiation of chondrocytes, causing hypertrophy, and enhances the commitment of mesenchymal cells to the chondrocyte lineage. Further, GDF5 stimulates proteoglycan synthesis in articular cartilage explants. The genetic analysis reported that GDF5 is a susceptibility gene for osteoarthritis (Miyamoto et al., 2007). Researchers demonstrated that rat models with GDF5 deficiency expressed abnormal morphology of the joint, including femoral length, bicondylar width of the femur, the width of the tibial plateau, width of the intercondylar notch, curvature radius of the medial femoral condyle, depth of the trochlear groove, trochlear groove sulcus angle, and other important joint parameters (Pregizer et al., 2018). But it is unclear whether GDF5 has any role in the knee beyond determining its shape or soft tissue composition. The susceptibility to OA may attribute to the abnormal morphology of the joint shape, resulting in the unbalanced mechanical loading. Decreased GDF5 levels in fully formed adult knees may also influence OA risk by impairing homeostasis in healthy joints or by accelerating degeneration due to injury. Recently, the intra-articular recombinant human GDF5 supplementation was reported to prevent and even reverse OA disease progression in the rat medial meniscus transection OA model (Parrish et al., 2017). The mechanism behind may be correlated to the pivot role for GDF5 during the establishment of hyaline cartilage and in the maintenance of the articular cartilage (McHugh, 2017). However, the exact reason underlying its treatment effect remains to be discovered. Other growth plate genes are also associated with the susceptibility of OA, including COL11A1, ANP32E, BMP5, and others (Reynard and Barter, 2020). However, their correlation with the osteochondral interface still needs to be addressed.

## Neurovascular Growth in the Osteochondral Junction of OA

Angiogenesis is necessary for bone growth. As the main source of oxygen, nutrients, hormones, neurotransmitters, and growth factors delivered to bone cells, the vasculature is essential for proper bone development, regeneration, and remodeling. However, the capillary vessels located at the channel in the healthy joint branches to the osteochondral interface but were rarely found to exceed the tidemark into hyaline cartilage (Woods et al., 1970; Suri et al., 2007; Walsh et al., 2007). Recent studies have shown that in OA, the fine neurovascular vessels were found in the subchondral bone plate and osteophytes. The nerves marked with the sensory and sympathetic cluster of differentiation were also found in these areas (Wojtys et al., 1990; Suri et al., 2007). Walsh et al. (2010) reported that in the channel that touches the tidemark, the general morphology of fibrovascular tissue is visible at the junction of the cartilage and the fibrosis, fissures at the articular surface. These α-actin-positive cells, represented in smooth muscles, could also be the microvascular tunnels in the subchondral bone. Positive blood vessels were also found within the subchondral space. CD34-positive endothelium, PCNA-positive nuclei, and 4′-6′-diamidino-2-phenylindole (DAPI) stained non-proliferating nuclei have been stained in the vascular cells in a vascular channel that crosses the tidemark. VEGF-, PDGF- or NGF-positive cells were also found in the vessel channels of subchondral bone (Pesesse et al., 2011). According to the previous findings, tidemark duplication was associated with neurovascular invasion. This process is usually related to the protrusions of CCZ to the deep part of the hyaline articular cartilage. However, whether the deeper extensions of blood vessels play a role of initiation or consequence of mineralization of the cartilage and advancement of the tidemark toward the surface remains the subject of ongoing research (Bullough and Jagannath, 1983; Oegema et al., 1997).

The cause of neurovascular invasion remains unclear. There are two commonly accepted mechanisms for the angiogenesis of OA in the osteochondral interface, including increased macrophage infiltration and reduced cartilage resistance to angiogenesis (Mapp and Walsh, 2012). An immunohistochemical study (Franses et al., 2010) showed that protease inhibitors and VEGF are predominantly in the chondrocytes located in the superficial layer of cartilage, are less common in the translational layer, and rare in the radiate layer. The concentration of vasculogenic factors, including VEGF, TIMP-1, TIMP-3, SLPI, and PAI-1, were all upregulated in OA samples compared to the healthy ones. Thus, these upregulated markers are correlated with the severe degradation of cartilage. Interestingly, the expression of VEGF, rather than the expression of the protease inhibitor, has a close relationship with denser vascularity. These intriguing facts revealed that the resistance of healthy cartilage relies more on its microenvironment rather than the regulation of the protease inhibitor. Although the upregulation of the protease inhibitor indeed alleviated the angiogenetic function of VEGF in the superficial layer of cartilage, chondrocytes that are located in the radiate layer are unable to express anti-angiogenic protease inhibitors that may cause blood vessels to invade the articular cartilage (Franses et al., 2010; Figure 2).

## Abnormal Molecular Exchange and Cellular Crosstalk Through the Osteochondral Interface

Westacott et al. (1997) hypothesized that an increase in subchondral activity can adversely affect cartilage metabolism. However, if this assumption was correct, it would require altered signaling molecules to be transported between the subchondral bone and the cartilage cavity (Westacott et al., 1997). Earlier studies reported that in the healthy knee joint of adult rabbits, substances did not virtually diffuse into the hyaline cartilage through CCZ and the subchondral bone. However, the number of pores of subchondral bone were increased in OA (Duncan et al., 1987; Clark and Huber, 1990; Li et al., 1999; Lyons et al., 2006; Sniekers et al., 2008). Histological sections of tartrate-resistant acid phosphatase (TRAP) staining showed that the area of osteoclast resorption located in subchondral bone extended to the CCZ, indicating chemotaxis induced movement of osteoclasts (Duncan et al., 1987). An *in vivo* fluorescent study also showed that multiple molecules could pass through the osteochondral interface, and their diffusion rate varies with the degree of mineralization and OA progression (Pan et al., 2009). Moreover, it was believed that microcracks are conducive to substance transport in OA. Hwang et al. (2008) used water as a medium and drew a similar conclusion concerning hydraulic conductance. The above findings indicate that the upregulated porosity is related to the cartilage infiltration of OA.

Subchondral bone is another important aspect of bone and cartilage signaling pathways, and its role in the progression of OA has been increasingly becoming the focus of researchers and clinicians. All cell types within this area, including osteoblasts, osteoclasts, osteocytes, and bone-lining cells, may have a certain degree of crosstalk with chondrocytes. Subchondral bone influences the overlying articular cartilage through both biomechanical and biochemical pathways. Several mechanisms have an impact on the osteochondral interface. By utilizing the photobleaching (FLIP) method, Pan et al. found that both hyaline cartilage degeneration and neurovascular invasion can increase crosstalk via the altered molecular exchange (Nakamura and Mizuno, 2010). The signal across the osteochondral junction can be exemplified by hepatocyte growth factor (HGF), an angiogenic factor that acts by phosphorylation of its receptor tyrosine-protein kinase Met (C-Met) (Nakamura and Mizuno, 2010). C-Met is not only expressed in the liver but also expressed by chondrocytes, in which HGF is a regulator that helps the excretion of proteoglycan and type II collagen (Pfander et al., 1999). HGF is always found in the CCZ and radial zone of hyaline cartilage in OA (Pfander et al., 1999; Reboul et al., 2001). However, it seems that it was only found in a specific subtype of chondrocytes with a truncated shape (Bullough and Jagannath, 1983). In contrast, the bioactive HGF was only found in osteoclasts in the cement line side of the subchondral bone plate. This phenomenon shows that cytokines transfer across the osteochondral interface to play a role.

An osteoblast-chondrocyte co-culture system can also provide insights into crosstalk between both cell types (Sanchez et al., 2005). Osteoblast and chondrocytes were cocultured in the environment without bone remodeling signals. The results showed that the osteoblasts in the subchondral bone induced a decreased aggrecan gene expression, as well as elevated gene expression of metalloproteinase-3 and -13 in chondrocytes. The co-culture results indicated that the OA chondrocytes altered the chondrocytes gene expression through crosstalk. Another example is the stromal cell-derived factor 1/chemokine receptor type 4 (SDF-1/CXCR4) axis. Qin et al. (2019) firstly revealed that the increase of SDF-1 functions as a degeneration accelerator through the recruitment of mesenchymal stem cells (MSCs) and osteoblasts in the OA mouse model. Furthermore, this increase of chemokine then crosses through the osteochondral interface to the superficial layer of hyaline cartilage. Finally, SDF-1 from subchondral bone bind to chemokine receptor type 4 (CXCR4) in chondrocytes at the superficial layer. The binding of SDF-1 and CXCR4 then induces the degeneration of articular chondroid-like kinase 5 by promoting the transformation of growth factor-beta receptor I from activin receptor-like kinase 5 to activin receptor-like kinase 1 in chondrocytes. Thus, the inhibition of this axis has therapeutic implications for a novel target in OA treatment.

## Therapeutic Implications Targeting Osteochondral Unit

### Targeting Chondrocyte Hypertrophy Signals

Chondrocyte hypertrophy is an essential feature for OA, and it is widely believed that the hypertrophic chondrocytes correlate with the progression of OA. Recently, multiple studies have focused on inhibiting chondrocyte hypertrophy as a target for OA treatment. Matrix metalloproteinase inhibitors (MMPs) are a family of zinc-dependent endopeptidases with more than 20 members, involved in multiple diseases, including OA. Several MMP-13 inhibitors were investigated for potential use in OA treatment. Although they showed good results in *in vitro* experiments, they have not been successful in clinical trials due to the heterologous effects and musculoskeletal syndrome (Li et al., 2011; Winer et al., 2018). Recent studies have been focusing on the topical use of MMP-13 inhibitors in the lesions to enhance the efficacy and limit its adverse effects (Jahangir et al., 2020). However, more clinical trials are needed to justify its therapeutic effects in humans.

RNA silencing of relevant genes is also under development by many researchers since it can specifically knock out or inhibit the expression of specific genes, thus blocking the transformation of the chondrocytes to the hypertrophic phenotype. There are mainly two tools for suppressing protein expression through gene silencing, including small interfering RNA (siRNA) and short hairpin RNA (shRNA). This process is achieved by selectively inactivating the corresponding mRNA of the target gene by double-stranded RNA (dsRNA). RNA interference is activated by double-stranded RNA transported into the cell cytoplasm. The silencing mechanism can lead to the degradation of target mRNA induced by siRNA or shRNA or the inhibition of specific mRNA translation induced by small RNA (miRNA). Short hairpin RNAs (shRNA) are reported to directly silence the hypertrophy-related genes, including RUNX2, CBFB, and other genes. siRNA targeting YAP is also used to prevent cartilage hypertrophy, and this involves the inhibition of the β-catenin (Yang et al., 2017; Gong et al., 2019). Despite the success of *in vitro* experiments, most RNA silencing treatment methods still face the challenge of finding a safe, efficient, and selective delivery pathway. Although new siRNA nanocarriers have been tested in clinical trials, there are still some challenges and multiple obstacles in RNA silencing therapy that need to be overcome (Chakraborty et al., 2017; Saw and Song, 2020).

### Targeting Angiogenesis and Pain Signals

Nerve growth factor (NGF), represented by neurotrophin, was primarily determined to support the survival, development, and functioning of neurons. The neurotrophin family includes neurotrophin 3 (NT-3), neurotrophin 4 (NT-4), and brain-derived growth factor (BDNF) (Bothwell, 2016; Schmelz et al., 2019). Among the potential novel strategies for pain control in OA (Malfait and Miller, 2016), the clinical development of the strategy for neurotrophin and nerve growth factor is the most advanced. Its therapeutic effect lies not only in its role as a growth factor for cells in the peripheral nervous system but as a critical mediator of acute and chronic pain. Different biological actions of NGF contribute to its pro-algesic effects, including NGF-induced sensitization of peripheral nociceptive terminals and NGF-induced sprouting of sensory nerves. Monoclonal antibodies can be used to inhibit the function of NGF lies in its high-affinity to homologous receptor, tropomyosin-related kinase (Trk) A, thereby blocking its biological activity. As a result, humanized monoclonal antibodies such as Tanezumab, Fullanumab, and Fasinumab, have been successfully developed by the pharmaceutical industry and have shown high efficacy in RCTs in pain management in OA patients (Miller et al., 2017). Systematic reviews detailing the efficacy of NGF- monoclonal antibodies in OA clinical trials have been published elsewhere (Schnitzer and Marks, 2015; Kan et al., 2016; Chen et al., 2017). Administration of NGF-Ab attenuates pain-related behavior in animal models (Ghilardi et al., 2012). Compared with age-matched asymptomatic controls, patients with symptomatic knee arthritis also showed increased neurofibril density in the synovium, elevated levels of NGF and TrkA (Kc et al., 2016). However, researchers unexpectedly discovered an osteonecrosis-like condition, which caused the FDA to shelve all experimental plans for NGF-Ab from 2010 to 2012 (Hochberg, 2015; Schmelz et al., 2019). The careful investigation revealed that most cases, which were first recognized as osteonecrosis, suffered from rapidly progressive OA. As the dose of tanezumab increases, the risk of rapidly progressive OA will gradually increase. Meanwhile, the combined use of tanezumab and NSAIDs and the presence of pre-existing subchondral insufficiency fractures can also increase the risk of rapidly progressive OA (Hochberg et al., 2016). Similar findings have been verified in studies of drug safety events in other NGF-Ab research (Hochberg, 2015). So far, the pathophysiology of rapidly progressive OA caused by NGF-Ab is poorly understood. The current possible mechanisms include neuropathic arthritis, analgesic arthritis, and pre-existing low bone integrity. However, the overall adverse event is quite low and NGF-Ab is well-tolerated for patients (Schnitzer and Marks, 2015). More studies should be done to investigate the mechanism behind the adverse effect to avoid this phenomenon.

Angiogenesis activators include VEGF Endoglin HGF IL-1, −8, −18, TGF-β_1__/__2__/__3_ TNF-α, CTGF, Substance P, PGE2, Nitric oxide, Histamine, FGF-2, FGF-1, ESAF, IGF-1, EGF, PDGF-A, Transferrin, Cyr61, and MMP-9/gelatinase B, while anti-angiogenic inhibitors include Thrombospondin-1, Leukemia Inhibitory Factor, TIMP-1 et -2, TGF-β, TNF-α, Chondrocyte inhibitor of angiogenesis, Chondromodulin-1, Troponin-I, and Thrombospondin-3 (Gerber and Ferrara, 2000). Any factors that inhibit activators or enhance inhibitors could be useful in the therapeutic process. Xufang et al. reported that the intra-articular injection of LV-VEGF shRNA would alleviate the progression of OA by inhibition of VEGF (Zhang et al., 2016). Another study used a local injection of bevacizumab, which showed reduced articular cartilage degeneration, osteophyte formation, and synovitis compared with intravenous administration and control group (Nagai et al., 2014). Different molecular targets are summarized in Figure 2.

## Future Directions

Studies describing the osteochondral interface are limited due to the ultra-thin structure and the difficulty in separating individual cells within the solid bone-cartilage layer. Hence its relationship with OA was obscure. However, with interdisciplinary research, many new techniques that were used for detecting nano-scale particles have been used to identify the relationship between the osteochondral interface and the progression of OA.

Future research should focus on understanding the etiology of the individual chondrocytes in the osteochondral interface. There is also a need for research that focused on the understanding of gene regulatory patterns at the commence of OA and its ultrastructure change, as well as cell crosstalk and cell subpopulation. With new techniques, including single-cell sequencing (Ji et al., 2019), laser scanning confocal microscope and laser capture and dissection microscope, proteomics, and other novel instruments and concepts, disturbances regarding gene and protein in extremely small scales can be identified. The new findings would give us to a deeper understanding of the importance of how the osteochondral interface change in phenotype, genotype, proteomics as well as its metabolism, and how the turbulence exacerbate the progression of the OA.

## Conclusion

The osteochondral interface undergoes multiple pathological changes during OA, and it includes morphological changes including tidemark duplication and roughness, thickening of CCZ, the occurrence of endochondral ossification, microcracks, neurovascular invasion, enhancement of perfusion, and elevated level of crosstalk through the osteochondral interface. A thorough understanding of these changes and mechanisms will support treatment approaches for this commonly occurring disease.

## Author Contributions

XW and IP: conception and design and drafting of the manuscript. XF, XW, and IP: collection and assembly of data. XF, XW, RC, YX, and IP: critical revision of the manuscript for important intellectual content. IP and YX: obtain of funding. All authors take responsibility for the integrity of the manuscript.

## Conflict of Interest

The authors declare that the research was conducted in the absence of any commercial or financial relationships that could be construed as a potential conflict of interest.
